# Relationship between LDL-cholesterol, small and dense LDL particles, and mRNA expression in a cohort of African Americans

**DOI:** 10.1152/ajpheart.00332.2024

**Published:** 2024-07-19

**Authors:** Ana Diallo, Malak Abbas, Gabriel Goodney, Elvin Price, Amadou Gaye

**Affiliations:** ^1^School of Nursing, Virginia Commonwealth University, Richmond, Virginia, United States; ^2^National Human Genome Research Institute, National Institutes of Health, Bethesda, Maryland, United States; ^3^Department of Pharmacotherapy and Outcomes Science, Virginia Commonwealth University, Richmond, Virginia, United States; ^4^Department of Integrative Genomics and Epidemiology, School of Graduate Studies, Meharry Medical College, Nashville, Tennessee, United States

**Keywords:** African American, dense LDL particles, LDL cholesterol, mRNA, small and dense LDL particles

## Abstract

Understanding the characteristics and behavior of low-density lipoprotein (LDL) particles provides insights into the atherogenic risk of elevated LDL cholesterol in hypercholesterolemia, cardiovascular disease risks. Studying LDL particles helps identify specific LDL subtypes [e.g., small dense LDL particles (sdLDL)] that may be atherogenic and, consequently, potential targets for therapeutics. This study cohort consists of African Americans (AAs), a population disproportionately affected by cardiovascular diseases, thereby accentuating the importance of the investigation. Differential expression (DE) analysis was undertaken using a dataset comprising 17,947 protein-coding mRNAs from the whole blood transcriptomes of 416 samples to identify mRNAs associated with low-density lipoprotein cholesterol (LDL-C) and sdLDL plasma levels. Subsequently, mediation analyses were used to investigate the mediating role of sdLDL particles on the relationship between LDL-C levels and mRNA expression. Finally, pathway enrichment analysis was conducted to identify pathways involving mRNAs whose relationship with LDL-C is mediated by sdLDL. DE analysis revealed 1,048 and 284 mRNA transcripts differentially expressed by LDL-C and sdLDL levels, respectively. Mediation analysis revealed that the associations between LDL-C and 33 mRNAs were mediated by sdLDL. Of the 33 mRNAs mediated by sdLDL, 18 were mediated in both males and females. Nine mRNAs were mediated only in females, and six were mediated only in males. Pathway analysis showed that 33 mRNAs are involved in pathways associated with the immune system, inflammatory response, metabolism, and cardiovascular disease (CVD) risk. In conclusion, our study provides valuable insights into the complex interplay between LDL-C, sdLDL, and mRNA expression in a large sample of AAs. The results underscore the importance of incorporating sdLDL measurement alongside LDL-C levels to improve the accuracy of managing hypercholesterolemia and effectively stratify the risk of CVD. This is essential as differences in sdLDL modulate atherogenic properties at the transcriptome level.

**NEW & NOTEWORTHY** The study investigated the interplay between LDL-C and mRNA expression, focusing on the role of small dense LDL (sdLDL) particles and sex differences. Differential expression analysis identified 1,048 and 284 mRNAs associated with LDL-C and sdLDL levels, respectively. Mediation analysis revealed that sdLDL mediates the relationship between LDL-C and 33 mRNAs involved in immune, inflammatory, and metabolic pathways. These findings highlight the significance of sdLDL in cardiovascular disease risk assessment and underscore sex-specific differences in lipid metabolism.

## INTRODUCTION

Managing low-density lipoprotein cholesterol (LDL-C) levels is crucial for hypercholesterolemia management and primary prevention of cardiovascular disease (CVD) risks (1). Despite strong evidence that lowering LDL-C levels effectively reduces CVD risks, hypercholesterolemia and CVD incidences remain high, particularly among African Americans (AAs) (2). In fact, African American women are less likely to be screened for elevated LDL-C and receive lipid-lowering therapies (3–5). Furthermore, many individuals, treated with lipid-lowering medications, still face challenges to control LDL-C levels and continue to experience significant CVD risks (6, 7). Therefore, a growing number of epidemiological studies support considering LDL particles, such as small dense LDL (sdLDL), as clinically useful metrics to manage hypercholesterolemia and cardiovascular disease (CVD) risks (8–11).

Small dense LDL particles are highly atherogenic and independent risk factors for CVD due to their prolonged circulation time, increased arterial wall penetration, and susceptibility to oxidative modifications associated with plaque formation and atherosclerosis (12). These properties make sdLDL proatherogenic (13), prothrombotic (14), and proinflammatory (15). Studies such as the Atherosclerosis Risk in Communities reported higher coronary heart disease incidence with increased sdLDL levels (16). Similarly, the Quebec Cardiovascular Study found sdLDL levels to be strong predictors of CVD (17). Variation in sdLDL levels is influenced by complex genetic and environmental factors. For instance, elevated triglycerides increase sdLDL through heightened cholesterol-ester transfer protein (CETP) activity and more triglyceride-rich lipoprotein cholesterol, leading to smaller, denser particles (18). Heritability also plays a role in LDL particle phenotypes, including sdLDL, ranging from 40 to 60% of variability (19). Moreover, recent evidence highlights the role of epigenetics, with higher methylation of CPT1A and ABCG1 genes associated with lower sdLDL levels (20). These evidences indicate that measuring sdLDL could offer additional predictive value for CVD risks (19, 21), especially among AAs, a population group disproportionally affected by suboptimal management of elevated LDL-C and CVD risks (5). Such knowledge can lead to improved personalized treatment strategies and advancements in the prevention and management of CVD.

Numerous studies have highlighted the roles of LDL-C and sdLDL-C in CVD, independently; but few have explored their relationship in humans. Vekic et al. (22) reported a strong link between sdLDL levels and the LDL B phenotype, which is characterized by smaller and denser LDL particles, emphasizing the need to consider particle concentration in CVD risk assessment. Bhattarai et al. (23) found a significant positive correlation between sdLDL and LDL-C levels in participants with coronary artery disease, type 2 diabetes, and obesity, underscoring the importance of assessing sdLDL alongside LDL-C for comprehensive CVD risk evaluation. Examining the interrelationships among LDL-C and sdLDL particles can uncover distinct mechanistic pathways that may independently confer cardiovascular risk.

In addition, there is a lack of information about sex differences in these relationships. Sex difference in lipid profiles is well documented (24–26). Women exhibit a more atherogenic lipid profile compared with men throughout various life stages, influenced by transitions like the menstrual cycle, pregnancy, breastfeeding, and menopause (27). After the age of 19, premenopausal women typically have a more favorable lipid profile compared with men (28). In the age groups of 20–39 and 40–65 yr olds, men exhibit a more atherogenic lipid profile, characterized by higher LDL-C and triglyceride levels, as shown by data from the Copenhagen General Population Study (29). Understanding the interrelationships between LDL-C and sdLDL and sex-specific differences could provide important insights into the atherosclerosis process, precursor of CVD outcomes, and help tailor more effective prevention strategies.

Transcriptome analysis allows for a comprehensive profiling of messenger RNA (mRNA) expression patterns involved in LDL metabolism, receptor interactions, and intracellular transport. Furthermore, mRNA expression profiles shed light on the genetic and molecular mechanisms involved in lipid metabolism, atherosclerosis, and cardiovascular health (30). The hypothesis suggesting a significant relationship between LDL-C, sdLDL particles, and mRNA expression in elevated LDL-C levels and CVD risks is grounded in the integration of epidemiological data and molecular biology research. Epidemiological studies have consistently linked elevated LDL-C and sdLDL particles with increased atherosclerosis and CVD risk (16, 31, 32). Advances in molecular biology have identified specific genes and regulatory networks in lipid metabolism and inflammation, modulated in hypercholesterolemia and atherosclerosis (33–35). The human transcriptome is a complex network of coding and noncoding RNAs with critical roles in CVD (36). In their comprehensive review, Charles et al. (37) concluded that transcriptomic analysis is a powerful method for uncovering the regulatory mechanisms underlying CVD. A mice study suggests that sdLDL can promote the development of atherosclerosis by regulating the activity of gene networks (38) involved in cholesterol metabolism and the development of atherogenic lesions. Combining lipid profiling with gene expression analysis offers a comprehensive understanding of the interrelationships between LDL-C and sdLDL levels in CVD risk, aiding in identifying at-risk individuals and developing targeted therapies.

Therefore, transcriptome studies can provide a holistic view of the interaction networks governing LDL-C and sdLDL. The aim of this study is to establish the relationship between mRNA expression and LDL-C levels and to determine if this relationship is mediated by sdLDL levels, using data from a cohort of AA adults. We leverage whole blood mRNA sequencing (mRNA-seq) data to investigate the relationship between LDL-C and mRNA expression and the mediating role of sdLDL in that relationship. The study also seeks to investigate whether the mediation effect differs by sex.

## MATERIAL AND METHODS

### Data Description

The data included in this project are from the Genomics, Environmental Factors and the Social Determinants of Cardiovascular Disease in African Americans Study (GENE-FORECAST), a research platform that establishes a multiomics systems biology approach amenable to the multidimensional characterization of health and disease in African Americans (AAs). GENE-FORECAST used a community-based sampling frame of self-identified, US-born, AA men and women (ages 21–65 yr old) recruited from the metropolitan Washington D.C. area. This study was approved by the National Institutes of Health’s Institutional Review Board.

A description of the baseline characteristics of the GENE-FOREAST subjects included in the analyses is outlined in Table 1. LDL-C concentration was assessed as part of the fasting blood chemistry panel (overnight fast and no alcohol consumption for 24 h) from plasma collected in Lithium Heparin tubes. Among the 416 individuals examined, only 41 (10%) were receiving lipid-lowering medications, while 101 (24%) exhibited LDL-C levels equal to or exceeding 129 mg/dL. The *NMR LipoProfile* (39) lipoprotein particle test was used to quantify LDL particles’ concentration. The particles are defined by size and density (sdLDL have a diameter <25.5 nm). In the study data, sdLDL is positively correlated with LDL-C. Age displayed a significant correlation with LDL-C levels, whereas factors such as sex, body mass index (BMI), and educational attainment did not. The study population consisted of a larger proportion of females. Over one-third of the individuals possess a graduate-level education or higher.

**Table 1.** Baseline characteristics of the 416 samples included in the differential expression analysis and their correlation with LDL-C

Characteristic	Mean or Count	SD or Proportion	Correlation with LDL-C	*P* Value of Correlation
LDL-C, mg/dL	105	33		
sdLDL, mg/dL	477	333	0.4	3.38e-17
Lipid-lowering medication			−0.1	0.04
No	376	90%		
Yes	41	10%		
Body mass index	32	7	0.07	0.13
Age, yr	48	12	0.17	7.00e-04
Sex			−0.01	0.85
Female	289	69%		
Male	127	31%		
Education			−0.05	0.31
≤high school	23	6%		
Some college/vocational/ technical school	64	15%		
College graduate	72	17%		
>Graduate	83	20%		
Not available	174	42%		

LDL-C, low-density lipoprotein-cholesterol; sdLDL, small and dense LDL.

The transcriptome data consisted of the messenger RNA sequencing (mRNA-seq) data of whole blood (buffy coat). Total RNA extraction was carried out using MagMAXTM for Stabilized Blood Tubes RNA Isolation Kit as recommended by the vendor (Life Technologies, Carlsbad, CA). For library preparation, total RNA samples are concentration normalized, and ribosomal RNA (rRNA) is removed. Pooled libraries are bound to the surface of a flow cell and each bound template molecule is clonally amplified up to 1,000-fold to create individual clusters. Illumina paired-end 100 base pair sequencing was performed on a HiSeq2000 analyzer (Illumina) with an average sequencing depth of 75 million reads per sample. The mRNA expression was quantified using a bioinformatics pipeline developed by the Broad Institutes and used by the Genotype-Tissue Expression (GTEx). The pipeline is detailed in the GitHub software development platform (40). Transcripts that did not achieve an expression of 1 read count per million (CPM) in at least three samples were excluded. The expression data were normalized using the trimmed mean of M-values (TMM), an optimal method for read count data (41). Principal component analysis was conducted to identify and exclude sample and gene outliers. After quality controls (QCs), 17,947 protein-coding mRNAs were included in the analysis.

### Statistical Analyses

**Figure 1.**
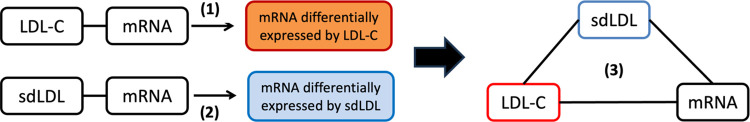
Overview of the analyses undertaken: *1*) the relationship between low-density lipoprotein-cholesterol (LDL-C) and mRNA expression was assessed; *2*) the relationship between small and dense LDL (sdLDL) and mRNA expression was then evaluated; and finally, *3*) mediation analysis was conducted to identify associations between LDL-C and mRNA that are mediated by sdLDL.

The analytical steps of the project are depicted graphically in Fig. 1 and detailed in the subsequent paragraphs.

#### Differential expression analysis.

Differential expression (DE) analysis was conducted on a set of 17,947 protein-coding mRNAs, using the R library *edgeR* (41). This algorithm fits a negative binomial model to the read counts of mRNAs and subsequently computes likelihood ratio tests for the coefficients within the model. The association was adjusted for age, sex, and level of education (a proxy for socioeconomic status). Statistical significance in differential expression between the upper and lower tertiles of LDL-C and sdLDL was determined based on a Benjamini–Hochberg false discovery rate (FDR) adjusted *P* value ≤ 0.05. The DE analysis focused on the extremes of the lipid variable distribution, specifically the upper and lower tertiles because the tails of the distribution can help identify mRNAs and pathways that are strongly associated with very high or very low LDL-C or sdLDL levels, which might be more pronounced and biologically relevant than associations found when examining the entire range of LDL-C/sdLDL levels. Furthermore, focusing on the tails of the distribution can increase the statistical power to detect differential expression because extreme groups are more likely to show clear differences, reducing the noise and variability that might obscure significant findings in our heterogeneous study population.

#### Mediation analysis.

This analysis was initiated to identify mRNAs wherein the association between mRNA and LDL-C is modulated by sdLDL. For sdLDL to qualify as a mediator in any mRNA-LDL-C relationship, three prerequisites must be satisfied: *1*) the mRNA-LDL-C association must be statistically significant after adjusting for age, sex, and multiple testing; *2*) the association between the mediator (sdLDL) and the mRNA must be statistically significant after adjusting for age, sex, and multiple testing; and *3*) the effect size of the mRNA-LDL-C association (expressed as log fold change or logFC) must decrease when the mediator is introduced into the model as a covariate.

Subsequent to the identification of mRNAs meeting the first two conditions (i.e., those differentially expressed by both LDL-C and the sdLDL), an additional series of differential expression analyses was executed, incorporating the mediator (sdLDL) as covariates into the model.

#### Pathway enrichment analysis.

Pathway enrichment analysis was performed to identify pathways enriched within the list of mRNAs whose association with LDL-C is mediated by sdLDL. This analysis was conducted using a function of the R library *clusterProfiler*, which runs hypergeometric tests, with mRNAs from our results mapped to known genes cataloged in the Kyoto Encyclopedia of Genes and Genomes (KEGG) database (42).

#### Sex difference in mRNA-LDL relationships mediated by sdLDL.

The relationships between mRNA expression and LDL-C mediated by sdLDL were analyzed separately in females and males. Mediation analysis was conducted within each sex group to determine the presence of mediation and to assess if the effect size change attributable to sdLDL differs between males and females. The mediation analysis, described earlier, was applied identically to both groups. Overlapping observations were then summarized graphically to provide a clear comparison between the sexes. Finally, pathway enrichment analyses were carried out to identify shared as well as pathways specific to each sex group.

## RESULTS

### Differential Expression Analyses

**Figure 2.**
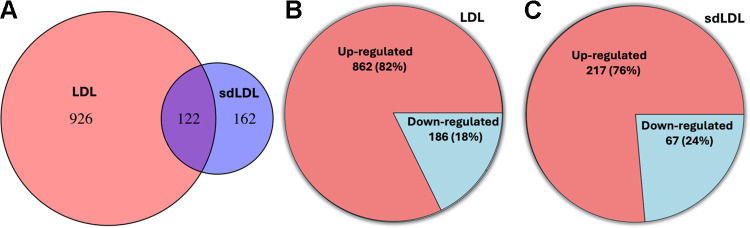
Summary of the differential expression analyses. *A*: overlap between mRNAs differentially expressed by low-density lipoprotein (LDL) and small and dense LDL (sdLDL). *B* and *C*: number of up- and downregulation mRNAs in the LDL analysis (*B*) and the sdLDL analysis (*C*).

Differential expression analysis was performed to identify mRNAs with different expression levels between the top and bottom tertiles of LDL-C and sdLDL. A total of 1,048 mRNAs were significantly differentially expressed with respect to LDL-C, and 284 mRNAs were significantly differentially expressed by sdLDL. Among these, 122 mRNAs were common to both LDL-C and sdLDL. The overlap between LDL and sdLDL and the number of u and downregulated mRNAs are reported graphically in Fig. 2. The full list of differentially expressed mRNAs is reported in Supplemental Table S1, *A* and *B* (all Supplemental tables are available at https://www.doi.org/10.6084/m9.figshare.26359408) for LDL-C and sdLDL, respectively.

Of the 122 overlapping mRNAs, 99 exhibited logFC in the same direction. Those 99 mRNAs were investigated in subsequent mediation analysis to assess the mediating role of sdLDL in the relationship between LDL-C and each mRNA. Table 2 reports the differential expression analysis results of a subset of 33 mRNAs associated with both LDL-C and sdLDL whose relationship with LDL-C is mediated by sdLDL.

**Table 2.** Diffrential expression analysis results of LDL-C and sdLDL for a set of 33 mRNAs whose association with LDL-C is mediated by sdLDL

mRNA	LDL-C			sdLDL		
	logFC	*P* Value	FDR Adjusted *P*	logFC	*P* Value	FDR Adjusted *P*
RNF182	−0.49	8.74e-18	2.61e-14	−0.34	3.88e-08	1.55e-05
TNS1	0.42	4.42e-17	8.81e-14	0.31	3.56e-09	2.00e-06
SLC12A1	−0.59	8.76e-17	1.57e-13	−0.28	1.10e-04	1.11e-02
ITGB3	0.40	3.43e-15	4.74e-12	0.20	1.02e-04	1.05e-02
RBM38	0.38	3.06e-14	3.43e-11	0.39	2.86e-13	3.95e-10
PF4	0.40	1.19e-13	1.02e-10	0.26	2.57e-06	5.01e-04
DMTN	0.38	1.46e-13	1.19e-10	0.23	1.96e-05	2.79e-03
NPRL3	0.40	2.08e-13	1.62e-10	0.29	3.22e-07	8.22e-05
MYOM2	0.43	4.24e-13	3.17e-10	0.31	2.23e-06	4.49e-04
FKBP8	0.33	3.68e-11	1.83e-08	0.25	1.64e-06	3.47e-04
RUNDC3A	0.34	1.78e-09	5.07e-07	0.40	5.33e-11	4.35e-08
RAD23A	0.36	2.29e-09	6.41e-07	0.25	8.51e-05	9.31e-03
TRIM58	0.30	2.40e-09	6.62e-07	0.28	1.85e-07	5.54e-05
BCL2L1	0.28	9.35e-09	2.12e-06	0.20	1.00e-04	1.04e-02
GUK1	0.29	1.01e-08	2.26e-06	0.19	4.78e-04	3.40e-02
E2F2	0.29	2.94e-08	5.80e-06	0.21	1.13e-04	1.13e-02
EVI2A	−0.29	5.00e-08	9.07e-06	−0.19	6.05e-04	4.08e-02
ASCC2	0.26	1.09e-07	1.76e-05	0.23	1.81e-05	2.63e-03
MRC2	0.31	1.27e-07	1.99e-05	0.24	1.73e-04	1.59e-02
MARCHF8	0.26	1.34e-07	2.07e-05	0.23	1.13e-05	1.78e-03
CARM1	0.31	1.74e-07	2.51e-05	0.21	6.90e-04	4.54e-02
CXCL5	0.31	2.49e-07	3.29e-05	0.25	8.74e-05	9.43e-03
TUBB2A	0.26	5.53e-07	6.65e-05	0.34	2.29e-09	1.37e-06
TMCC2	0.28	3.04e-06	2.58e-04	0.30	2.36e-06	4.66e-04
DCAF12	0.23	3.83e-06	3.07e-04	0.19	2.67e-04	2.24e-02
KLF1	0.26	5.75e-06	4.30e-04	0.24	7.39e-05	8.44e-03
HBQ1	0.52	9.17e-06	6.25e-04	0.56	4.49e-06	8.14e-04
ATOSB	0.23	1.91e-05	1.11e-03	0.23	8.78e-05	9.43e-03
DNAJA4	0.23	2.21e-05	1.24e-03	0.24	6.62e-05	7.76e-03
GYPB	0.21	2.25e-04	7.56e-03	0.24	4.64e-05	5.78e-03
BLVRB	0.18	2.52e-04	8.18e-03	0.23	2.10e-05	2.97e-03
ROGDI	0.21	1.29e-03	2.77e-02	0.25	3.97e-04	3.02e-02
IL4R	0.16	1.46e-03	3.01e-02	0.20	1.81e-04	1.65e-02

LDL-C, low-density lipoprotein-cholesterol; sdLDL, small and dense LDL; logFC, effect size; FDR, false discovery rate.

### Mediation Analyses

Mediation analysis was carried out for each of the 99 mRNAs associated with both LDL-C and sdLDL. We identified 33 mRNA-LDL-C associations mediated by sdLDL presented graphically in Fig. 2. The inclusion of the mediator (sdLDL) in the negative binomial model significantly attenuates the effect size (logFC), highlighting the influence of sdLDL on the relationship between LDL-C and mRNA. The mediation analysis’ logFC and *P* values are reported in Supplemental Table S1*C.* Three of the mRNAs mediated by sdLDL are downregulated in subjects with high LDL-C. The presence of sdLDL accounts for a proportion of logFC ranging from 55 to 95%, indicating nearly complete mediation for two of these mRNAs (SLC12A1, RNF182). A total of 30 mRNAs mediated by sdLDL are upregulated in subjects with high LDL-C. The presence of sdLDL accounts for 3% to 75% of the association between LDL-C and mRNA. For 12 of the mRNAs including TNS1, ITGB3, PF4, DMTN, NPRL3, MYOM2, RAD23A, BCL2L1, GUK1, E2F2, MRC2, and CARM1, sdLDL accounts for 50% or more of the logFC of mRNA expression between the high and low LDL-C groups.

### Pathways Enrichment Analysis

Enrichment analysis was conducted to identify canonical pathways enriched in the list of the 33 mRNAs whose relationship with LDL is mediated by sdLDL (Table 3). A total of eight pathways including some relevant to lipid metabolism were identified in the set of 33.

**Table 3.** Canonical pathways enriched in the list of mRNA whose relationship with LDL-C is mediated by sdLDL

Pathway ID	Subcategory	Description	*P* Value	mRNA
hsa04145	Transport and catabolism	Phagosome	0.002	ITGB3, MRC2, TUBB2A
hsa04640	Immune system	Hematopoietic cell lineage	0.014	ITGB3, IL4R
hsa04061	Signaling molecules and interaction	Viral protein interaction with cytokine and cytokine receptor	0.014	PF4, CXCL5
hsa04137	Transport and catabolism	Mitophagy	0.015	FKBP8, BCL2L1
hsa04060	Signaling molecules and interaction	Cytokine-cytokine receptor interaction	0.015	PF4, CXCL5, IL4R
hsa04151	Signal transduction	PI3K-Akt signaling pathway	0.026	ITGB3, BCL2L1, IL4R
hsa04630	Signal transduction	JAK-STAT signaling pathway	0.036	BCL2L1, IL4R
hsa04062	Immune system	Chemokine signaling pathway	0.047	PF4, CXCL5

LDL-C, low-density lipoprotein-cholesterol; sdLDL, small and dense LDL; PI3K, phosphoinositide 3-kinase.

### Sex Difference in mRNA-LDL Relationships Mediated by sdLDL

**Figure 3.**
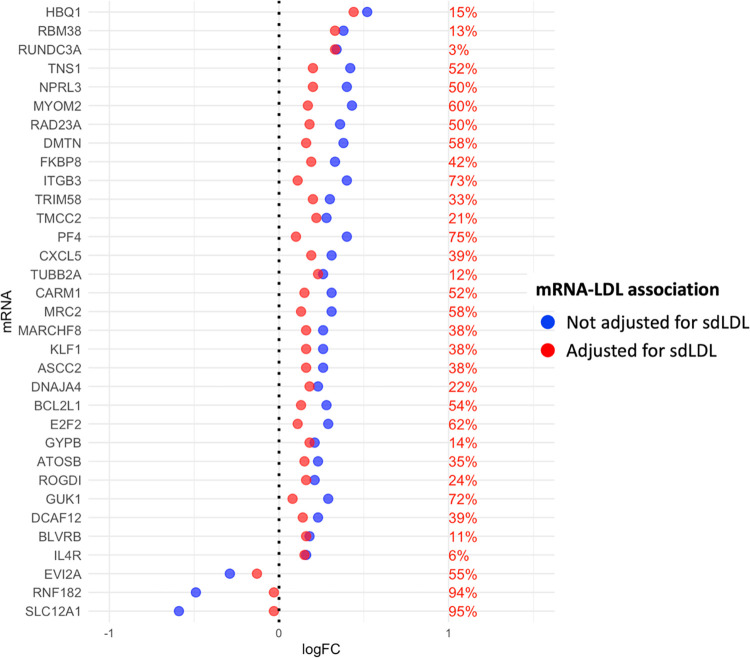
Graphical summary of the mediation analysis shows 33 mRNAs whose relationship with low-density lipoprotein-cholesterol (LDL-C) is mediated by small and dense LDL (sdLDL). Percent values in red represent the proportion of effect size (logFC) change attributable to sdLDL.

**Figure 4.**
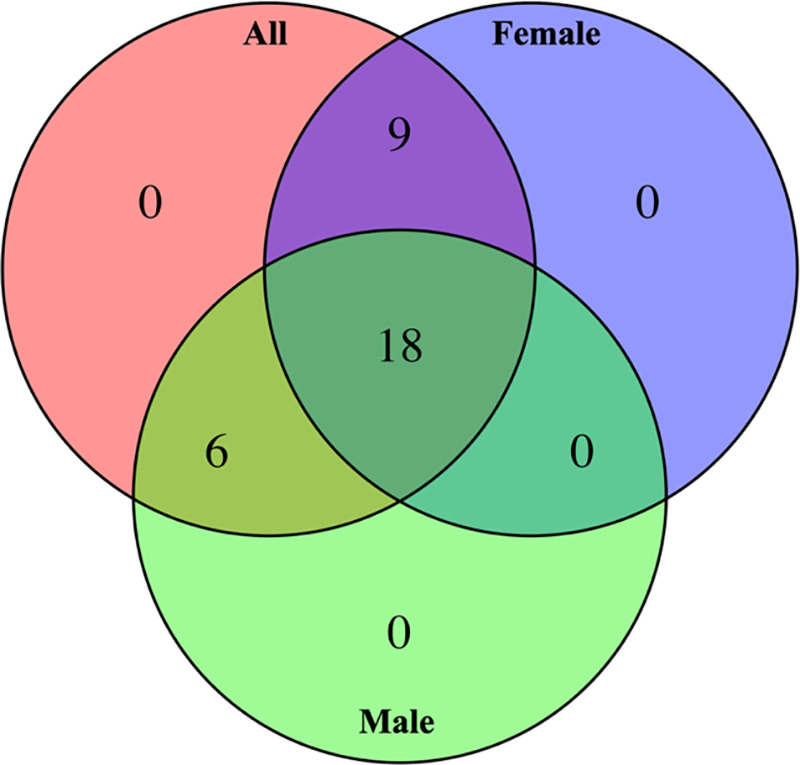
Number of mRNAs whose relationship with low-density lipoprotein-cholesterol (LDL-C) is mediated by small and dense LDL (sdLDL). Total of 18 mRNAs are mediated by sdLDL in all three analyses, while mediation is observed in nine mRNAs exclusively in females and six mRNAs exclusively in males.

**Figure 5.**
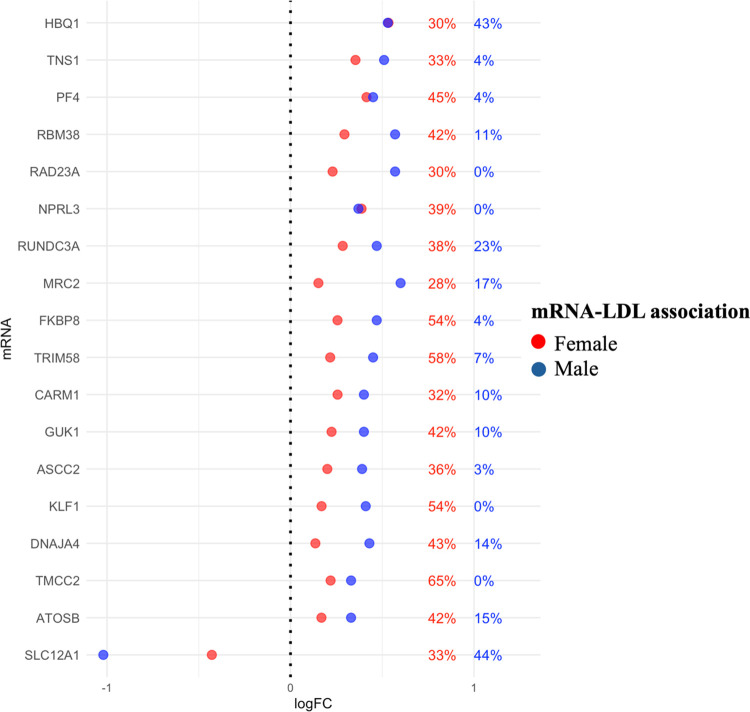
Differential expression of 18 mRNAs by low-density lipoprotein-cholesterol (LDL-C) in females and males. The percentages indicate the proportion of effect size (logFC) attributable to small and dense LDL (sdLDL) mediation. When only one point is shown, it means the logFC values for females and males are identical and overlap.

After the analysis with the entire dataset (summarized in Fig. 3), mediation analysis was also performed separately for females and males across the 33 mRNAs whose relationship with LDL-C was mediated by sdLDL in the entire dataset. The overlap between the whole dataset (females and males combined), females only, and males only is summarized in Fig. 4. Of the 33 mRNAs mediated by sdLDL, 18 showed mediation in both female and male groups. Nine mRNAs (RNF182, ITGB3, CXCL5, DMTN, EVI2A, MARCHF8, BCL2L1, E2F2, and IL4R) were mediated only in females, and six mRNAs (TUBB2A, MYOM2, DCAF12, ROGDI, GYPB, and BLVRB) were mediated only in males. Figure 5 illustrates the association between the 18 mRNAs and LDL-C in both females and males and shows the proportion of change in effect size (logFC) attributable to sdLDL, indicating a generally larger contribution of sdLDL to the mRNA-LDL-C relationship in females.

Pathway enrichment analyses were performed to identify significantly enriched pathways within the 18, 9, and 6 mRNAs mediated in the entire dataset, females only, and males only, respectively. Detailed results of the statistically significant enriched pathways (*P* value ≤ 0.05) are presented in Supplemental Table S1*D*. The specific pathways enriched in each group are summarized in Table 4.

**Table 4.** Pathways enriched in the lists mRNAs mediated by sdLDL in the full dataset, females only, and males only

Full Dataset (18 mRNA)	Females Only (9 mRNA)	Males Only (6 mRNA)
• PI3K-Akt signaling pathway	• PI3K-Akt signaling pathway • Pancreatic cancer • Chronic myeloid leukemia • Small cell lung cancer • Hematopoietic cell lineage • JAK-STAT signaling pathway • Hepatocellular carcinoma • Human T-cell leukemia virus 1 infection • Human cytomegalovirus infection • Cytokine-cytokine receptor interaction • MicroRNAs in cancer • Apoptosis - multiple species • Bladder cancer • Inflammatory bowel disease • Melanoma • Non-small cell lung cancer • Platinum drug resistance • p53 signaling pathway • Glioma • Pertussis • EGFR tyrosine kinase inhibitor resistance • Arrhythmogenic right ventricular cardiomyopathy • ECM-receptor interaction	• Porphyrin metabolism • Malaria • Gap junction

sdLDL, small and dense low-density lipoprotein; PI3K, phosphoinositide 3-kinase; EGFR, epidermal growth factor receptor; ECM, extracellular matrix.

## DISCUSSIONS

### Summary

This project delved into the interplay between LDL-C and mRNA expression, with a specific focus on delineating the distinct contributions of small and dense LDL particles (sdLDL) and the role of sex in the relationship. A comprehensive transcriptome analysis encompassing 17,947 protein-coding mRNA transcripts derived from 416 blood samples was conducted. The findings revealed that LDL-C and sdLDL concentrations were associated with the expression of 1,048 and 284, respectively.

Through mediation analysis, we observed that sdLDL wields significant influence over the relationship between LDL-C and 33 mRNAs. Pathway analyses further unveiled that the 33 mRNAs are integral components of pathways primarily associated with the immune system, inflammatory response as well as lipid metabolism.

These outcomes underscore the nuanced yet consequential impact of sdLDL on mRNA regulation. Furthermore, they substantiate the notion that LDL particles exert a distinctive and potent influence on mRNA pivotal to lipid metabolism and the pathogenesis of atherosclerosis, an important precursor of cardiovascular diseases.

### sdLDL Influence on mRNA Expression Pathways Related to LDL-C in Atherosclerosis

Small LDL particles exhibit increased atherogenicity attributed to their ability to permeate the arterial wall and undergo oxidative modifications, fostering inflammation and plaque formation (43). Their prolonged circulation time, stemming from a lower affinity for LDL receptors, accentuates the risk of cardiovascular diseases, emphasizing the critical need to manage elevated levels of sdLDL, especially in individuals predisposed because of genetic or metabolic factors (44, 45).

Several mRNAs involved in pathways enriched in the set influenced by sdLDL, including ITGB3, PF4, and CXCL5, have been previously implicated in cellular functions and their response to sdLDL levels (46, 47). Notably, ITGB3 and another mRNA of the same family ITGB2 play pivotal roles in integrin cell adhesion complexes and are integral to essential pathways, including cytokine receptor pathway, as well as the PI3K-Akt and chemokine signaling pathways (48, 49). These pathways assume significance in the context of atherosclerosis development, which often starts at sites of endothelial damage where sdLDL particles accumulate and breach the endothelial barrier (50). This triggers an inflammatory cascade, attracting immune cells into the vessel wall and facilitating the formation of foam cells from monocytes, a critical step in plaque development (51). Moreover, the migration of medial smooth muscle cells to the intimal layer and their collagen production represent crucial aspects of atherosclerotic plaque stabilization (52). However, this process may be compromised by the accumulation of a lipid-rich necrotic core, influenced by sdLDL and the activity of cytokine signaling pathways, such as IL-17 signaling (53), one of the pathways enriched in the set of genes whose relationship with LDL-C was mediated by sdLDL. These insights into gene expression changes associated with sdLDL contribute to a deeper understanding of the molecular mechanisms through which these particles may contribute to atherogenesis.

Key players in cell adhesion and signaling, such as ITGB3 and ITGA2B, play fundamental roles in cellular responses within atherogenic environments (54). Their influence extends to macrophage recruitment and the integrity of evolving plaques, implicating them in both the inflammatory response and the structural development of atherosclerotic lesions (55). Furthermore, genetic variants at the ITGB3 locus have been associated with plasma triglyceride levels, emphasizing the need for further exploration of the role of ITGB3 in lipid metabolism (56). This is particularly pertinent given the observed association in populations with β_3_-deficient Glanzmann’s Thrombasthenia (57), suggesting a broader and more complex influence of ITGB3 on cardiovascular health.

In our results, sdLDL accounts for 73% of the effect of LDL-C on ITGB3. ITGB3 encodes the integrin β_3_-subunit, a protein involved in platelet aggregation and endothelial function, processes that are crucial in the development of atherosclerosis. Given, the important role of ITGB3, our findings underscore the importance of sdLDL particles in cardiovascular disease mechanisms, highlighting their role in exacerbating the harmful effects of elevated LDL-C levels.

### Mediating Role of sdLDL Particles in the Function of CXCL5, in Atherosclerosis

CXCL5, also known as epithelial-derived neutrophil-activating peptide 78 (ENA-78), is a member of the CXC chemokine family which has an important role in immune responses, primarily by recruiting neutrophils to sites of inflammation. CXCL5 binds to its receptor, CXCR2, leading to the activation and recruitment of neutrophils to inflammatory sites. In atherosclerosis, oxidized LDL (ox-LDL) particles and other inflammatory stimuli in the arterial wall trigger the release of CXCL5, which recruits neutrophils to the endothelium. These neutrophils release enzymes and reactive oxygen species (ROS) that further damage the endothelial cells, promote the migration of monocytes into the intima, and facilitate the formation of foam cells from monocytes.

A review by Lu et al. (58) explored the role of chemokines such as CXCL5 in CVD and cited evidence for the protective role of CXCL5 with an inverse relationship between CXCL5 plasma levels and the severity of coronary artery disease (CAD). In addition, aggregates of CXCL5 and its receptor CXCR2 have been detected in coronary atherosclerotic plaques, in a way that implies a protective role for CXCL5 in CAD (59). Animal studies have provided results suggesting that CXCL5 may slow the progression of atherosclerosis by preventing the accumulation of macrophages and the formation of foam cells (60).

Our finding that sdLDL particles largely mediate the effect of LDL-C on CXCL5 expression highlights the important impact of these particles on molecular mechanisms underpinning CVD. Since CXCL5 is known to slow the progression of atherosclerosis, strategies that target sdLDL particles could enhance the protective effects of CXCL5, offering potential benefits for the management and treatment of atherosclerotic CVD.

### Sex Difference in the LDL-mRNA Relationships Mediated by sdLDL

The relationship between 18 mRNA and LDL-C was mediated by sdLDL in both males and females. Pathway analysis revealed the PI3K-Akt signaling pathway as enriched in the set of 18 mRNAs. PI3K-Akt signaling pathway is involved in cell survival, growth, and metabolism. This pathway is activated by growth factors, hormones, and other extracellular signals that bind to receptor tyrosine kinases (RTKs) on the cell surface, leading to the activation of PI3K (phosphoinositide 3-kinase). Activated PI3K then generates PIP3 (phosphatidylinositol 3,4,5-trisphosphate), which recruits Akt (also known as protein kinase B) to the cell membrane, where it is phosphorylated and activated. The PI3K-Akt signaling pathway plays a significant role in lipid metabolism by influencing the synthesis, storage, and breakdown of lipids (61, 62). PI3K-Akt signaling pathway is related to hypercholesterolemia, and particularly elevated LDL-C, through its influence on cholesterol homeostasis. PI3K-Akt signaling pathway influences cholesterol homeostasis through several mechanisms including the enhancing action of Akt on SREBP activity, which leads to increased cholesterol synthesis and the upregulation of LDLR expression by Akt, which facilitates the clearance of LDL from the bloodstream (LDL uptake). Furthermore, the PI3K-Akt pathway also modulates inflammatory responses in vascular cells; chronic inflammation is known to exacerbate atherosclerosis (63, 64). These evidences might explain why the PI3K-Akt pathway is crucial in both sexes in the relationship between mRNA expression and LDL-C levels through sdLDL and highlights its fundamental role in cardiovascular health and lipid metabolism.

In females, the mediation effect of sdLDL on the mRNA-LDL-C relationship involves a broad range of pathways, including various cancer pathways, JAK-STAT signaling, and cytokine interactions, reflecting a complex interplay of lipid metabolism, immune response, and cell signaling mechanisms specific to females. This phenomenon can be explained by several factors including hormonal influence and immune response difference in females. Estrogen, a predominant female hormone, has significant effects on lipid metabolism, inflammatory responses, and immune function (16, 65). Estrogen has been shown to influence the expression of various genes involved in these pathways, which may explain why these pathways are more prominent in females. Estrogen can modulate the activity of the PI3K-Akt and JAK-STAT pathways, thereby affecting cell survival, proliferation, and inflammation (66). Estrogen influences lipid profiles by increasing HDL cholesterol and reducing LDL cholesterol levels (67). This hormone also affects the composition and size of LDL particles, making them less atherogenic (68). The hormonal regulation may contribute to the observed sex differences in the mRNA-LDL-C relationship mediated by sdLDL. In addition, females typically exhibit stronger immune responses compared with males, which can be attributed to both genetic and hormonal differences. This heightened immune response can lead to differences in cytokine interactions and inflammatory pathways, such as those mediated by the JAK-STAT signaling pathway. The differences in immune regulation can result in sex-specific expression of genes involved in lipid metabolism, hypercholesterolemia, and atherosclerosis (69).

In males, the pathways involved in the mediation effect of sdLDL on the mRNA-LDL-C relationship are distinct and include porphyrin metabolism, malaria, and gap junctions, indicating a more specialized set of processes potentially related to energy metabolism, cell communication, and response to infections. Hormonal influence and energy metabolism may explain the sex-specific involvement of these pathways. Testosterone, the predominant male hormone, has significant effects on metabolism, including lipid metabolism and immune responses. It influences the expression of genes involved in energy production, cell signaling, and immune function. Testosterone can modulate pathways such as porphyrin metabolism, which is crucial for heme synthesis and energy production in cells (70). This hormonal regulation might explain why pathways related to energy metabolism are more prominent in males. Porphyrin metabolism is essential for the production of heme, a component of hemoglobin and various enzymes involved in oxidative metabolism (71, 72). Males typically have higher muscle mass and metabolic rates, which require efficient energy production systems. The involvement of porphyrin metabolism suggests a greater emphasis on maintaining energy homeostasis and metabolic function in males (73, 74). One of the six mRNAs specifically mediated by sdLDL in males only, BLVRB (Biliverdin Reductase B) is directly involved in heme metabolism, specifically in the reduction of biliverdin to bilirubin. This process is part of the broader porphyrin metabolism pathway, which is crucial for the production and breakdown of heme. Efficient heme metabolism is essential for maintaining redox balance and protecting cells from oxidative stress, which is a key factor in the development of atherosclerosis.

### Relevance for the African American Population

The study used a significant sample size of AAs, a demographic disproportionately impacted by suboptimal management of hypercholesterolemia, and CVD risks and often inadequately represented in existing genomic datasets. The elucidation of specific mRNAs and pathways influenced by sdLDL provides valuable insights into the molecular mechanisms underlying the heightened cardiovascular risk in African Americans. By highlighting the role of sdLDL in the regulation of key mRNAs and pathways related to lipid metabolism and inflammation, our research underscores the need for tailored therapeutic strategies that address the unique genetic and metabolic profiles of AAs. These findings advocate for more inclusive research efforts and the development of precision medicine approaches to improve screening and management of hypercholesterolemia as well as cardiovascular health outcomes in this vulnerable population.

### Strengths, Limitations, and Further Work

The analysis used high-throughput sequencing technologies for an unbiased transcriptome-wide examination of whole blood mRNA sequencing data. Whole blood transcriptome analysis provides a comprehensive view of mRNA expression throughout the entire body because it captures systemic responses and reflects the collective activity of various tissues and cell types.

Although our research provides valuable insights, it is imperative to recognize its limitations. The complexity of LDL-C metabolism suggests the influence of numerous factors, such as diet, that extend beyond the confines of this study. The transcriptome data reflect mRNA expression in whole blood, which does not provide the specificity of mRNA expression in specific organs and tissues that might be more relevant for LDL-C metabolism. The cross-sectional nature of the study design limits our capacity to infer causation. Longitudinal studies are essential to establish the temporal connection between LDL-C and mRNA expression variations and the influence of sdLDL levels over time. The results presented require validation through experimental studies.

### Conclusions

The study findings advocate for the inclusion of sdLDL particle measurement, in addition to LDL-C levels, to enhance the precision of hypercholesterolemia screening and management and to accurately stratify cardiovascular disease (CVD) risk because variations in sdLDL particle levels may confer differences in atherogenic properties (17, 75). Exploring the subclasses of LDL-C and circulating lipoproteins, characterized by their size and density, has the potential to refine approaches to hypercholesterolemia management and foster the development of targeted therapeutic strategies.

Prior investigations into the metabolic pathways regulating cholesterol homeostasis have significantly advanced therapeutic strategies. Although current lipid-lowering agents, particularly statins and fibrates, have demonstrated the capacity to alter LDL size, the effect varies among these agents. Consequently, a deeper exploration of the mediating role of LDL particles in the effect of LDL-C at the transcriptome level holds promise for characterizing the involvement of lipoprotein particles in cholesterol metabolic disorders and CVD. Such investigations could offer valuable insights into the development of personalized therapeutic interventions.

Finally, our findings underscore the importance of considering sex-specific biological mechanisms when studying the relationship between lipid metabolism and cardiovascular health. Understanding these differences can lead to more tailored and effective therapeutic strategies for managing hypercholesterolemia and reducing cardiovascular risks in both sexes.

## DATA AVAILABILITY

Data presented in this article cannot be publicly shared yet because of privacy restrictions. Requests to access the datasets should be directed to the corresponding author.

## SUPPLEMENTAL MATERIAL

10.6084/m9.figshare.26359408 Supplemental Table S1: https://www.doi.org/10.6084/m9.figshare.26359408.

## GRANTS

This research was supported by the Intramural Research Program of the National Human Genome Research Institute at the National Institutes of Health.

## DISCLAIMERS

During the preparation of this work, the authors used chatGPT to check and correct language spelling and grammar. After using this tool/service, the authors reviewed and edited the content as needed and take full responsibility for the content of the publication.

## DISCLOSURES

No conflicts of interest, financial or otherwise, are declared by the authors.

## AUTHOR CONTRIBUTIONS

A.G. conceived and designed research; A.G. analyzed data; A.D., M.A., and A.G. interpreted results of experiments; A.G. prepared figures; A.D., M.A., and A.G. drafted manuscript; A.D., M.A., G.G., E.P., and A.G. edited and revised manuscript; A.D., M.A., G.G., E.P., and A.G. approved final version of manuscript.
